# Species-specific markers provide molecular genetic evidence for natural introgression of bullhead catfishes in Hungary

**DOI:** 10.7717/peerj.2804

**Published:** 2017-02-28

**Authors:** Beatrix Béres, Dóra Kánainé Sipos, Tamás Müller, Ádám Staszny, Milán Farkas, Katalin Bakos, László Orbán, Béla Urbányi, Balázs Kovács

**Affiliations:** 1 Department of Aquaculture, Szent István University, Gödöllő, Hungary; 2 Regional University Center of Excellence in Environmental Industry, Szent István University, Gödöllő, Hungary; 3 Department of Environmental Safety and Ecotoxicology, Szent István University, Gödöllő, Hungary; 4 Reproductive Genomics Group, Temasek Life Sciences Laboratory, Singapore; 5 Centre for Comparative Genomics, Murdoch University, Murdoch, Australia

**Keywords:** *Ameiurus*, Species-specific marker, Introgression, Hybridization, Bullhead catfishes, Multiplex PCR

## Abstract

Since three bullhead catfish species were introduced to Europe in the late 19th century, they have spread to most European countries. In Hungary, the brown bullhead (*Ameiurus nebulosus*) was more widespread in the 1970s–1980s, but the black bullhead (*Ameiurus melas*) has gradually supplanted since their second introduction in 1980. The introgressive hybridization of the two species has been presumed based on morphological examinations, but it has not previously been supported by genetic evidence. In this study, 11 different Hungarian habitats were screened with a new species-specific nuclear genetic, duplex PCR based, marker system to distinguish the introduced catfish species, *Ameiurus nebulosus, Ameiurus melas*, and *Ameiurus natalis*, as well as the hybrids of the first two. More than 460 specimens were analyzed using the above markers and additional mitochondrial sequence analyses were also conducted on >25% of the individuals from each habitat sampled. The results showed that only 7.9% of the specimens from two habitats belonged to *Ameiurus nebulosus*, and 92.1% were classified as *Ameiurus melas* of all habitats, whereas the presence of *Ameiurus natalis* was not detected. Two specimens (>0.4%) showed the presence of both nuclear genomes and they were identified as hybrids of *Ameiurus melas and Ameiurus nebulosus*. An additional two individuals showed contradicting results from the nuclear and mitochondrial assays as a sign of a possible footprint of introgressive hybridization that might have happened two or more generations before. Surprisingly, the level of hybridization was much smaller than expected based on the analyses of the North American continent’s indigenous stock from the hybrid zones. This phenomenon has been observed in several invasive fish species and it is regarded as an added level of complexity in the management of their rapid adaptation.

## Introduction

Signs of natural hybridization and introgression between species can be detected in many taxonomic groups (Rieseberg, 1997; Argue & Dunham, 1999) as 25% of plants and about 10% of animals are capable of forming interspecies hybrids (Mallet, 2005). This process may have several evolutionary and ecological consequences such as the merging of taxonomical groups which may lead to the appearance of new, reproductively isolated hybrid species, or to the transmission of characteristics important for adaptation which happen through introgression (Arnold & Martin, 2009). In some cases, this phenomenon may be linked to the invasion of species or to their human-mediated introduction (José Madeira, Gómez-Moliner & Barbe, 2005). The latter influences populations of freshwater fish to a considerable level (Ferguson, 1990; Cross, 2000). Introgression changes the genetic background of the invasive species involved in hybridization, which may influence the adaptive spreading and the distribution of the species.

This phenomenon has been observed for the bullhead catfish species (genus: *Ameiurus*) in Europe. Originally three species, the brown, the black, and the yellow bullheads (*Ameiurus nebulosus, Ameiurus melas*, and *Ameiurus natalis*; formerly all three listed in the *Ictalurus* genus) were introduced to the continent (Carlander, 1977; Walter et al., 2014). These species are native to the North American continent and were first introduced into France from the Mississippi Basin in 1871 (Spillmann, 1961; Vinginder, 2003) for fish ponds and aquaria. In 1885, further shipments arrived in Germany as well and subsequent introductions may also have taken place since then (Pintér, 1976; Rutkayová et al., 2013).

Within a few decades, the bullheads spread to most European countries with the exception of the British Isles (Tortonese, 1970; Wilhelm, 1999). Until now, *Ameiurus nebulosus* has been detected in 23 European countries, whereas *Ameiurus melas* in 18 of those countries so far, but it continues to spread. The first specimens were noticed in Romania in 1997 (Wilhelm, 1998), in Slovakia in 1999 (Koščo et al., 2000), in Portugal in 2002 (Gante & Santos, 2002), in the Czech Republic in 2003 (Lusk, Luskova & Hanel, 2010), in new waters of Serbia in 2005 (Cvijanović, Lenhardt & Hegediš, 2005) and in the Iberian Peninsula (Garcia-De-Lomas et al., 2009). It was found in Poland in 2007, although it had probably been introduced into the country already in the late 19th century (Nowak et al., 2010), while the already present *Ameiurus nebulosus* continued to spread farther north (Kapusta et al., 2010). The presence of *Ameiurus nebulosus* and black bullheads in the Transcarpathian region of Ukraine was demonstrated in 2014 by a detailed morphological analysis (Movchan, Talabishka & Velikopolskiy, 2014). The *Ameiurus natalis* appeared and formed European populations only in Italy after it was introduced in 1906 (Wilhelm, 1999; Rutkayová et al., 2013).

The *Ameiurus nebulosus* and *Ameiurus melas* were first introduced into Hungary in 1902; and *Ameiurus nebulosus* soon became more widespread species (Wilhelm, 1999). After that, *Ameiurus melas* was re-introduced from Italy in 1980 (Harka, 1997) and started to spread (Pintér, 1991). According to subsequent fish-faunistic observations in Hungary, *Ameiurus melas* has gradually supplanted *Ameiurus nebulosus* in natural waters (Györe & Sallai, 1998; Sallai, 2000). With the spreading of the *Ameiurus melas*, the introgressive hybridization of the two species, based on morphological examination, was also assumed (Harka & Pintér, 1990). However, the morphological identification of such hybrids, or even the pure species proved to be challenging, even for experienced, and well-trained taxonomists (Rutkayová et al., 2013). Phenotypic traits have long been used to classify organisms (Lindsey, 1963; Specziár, Bercsényi & Müller, 2009; Rutkayová et al., 2013), but later the morphometry (Rohlf, 1990) and the geometric morphometry (Ibañez, Cowx & O’Higgins, 2007) offered more detailed method for identification of species and hybrid specimens. However, these methods are influenced by environmental (Jørgensen et al., 2008) factors as well as individual differences (Nowak et al., 2010). A more reliable identification is possible with molecular genetic markers. Mitochondrial sequence analyses (Müller et al., 2010), enzyme polymorphism (Poteaux, Berrebi & Bonhomme, 2000), PCR-RFLP (Müller et al., 2012), VNTR-s (Lu, Basley & Bernatchez, 2001), and AFLP (Scotti-Saintagne et al., 2004) were used as well as SNP-s (Glover et al., 2013) for species and hybrid identification in different fish species.

In the case of bullhead catfishes only the phenotypic traits (Hardman & Page, 2003; Rutkayová et al., 2013), mitochondrial sequence and PCR-RFLP marker were used in the past (Hardman & Page, 2003; Hunnicutt, Cingolani & Voss, 2005).

The aim of the present research was to examine the distribution and the level of potential hybridization of the *Ameiurus* species in Hungarian waters. For this purpose, species-specific genomic DNA markers were isolated and optimized to form a duplex PCR-based test for molecular identification of the three species and their potential hybrids. Together with mitochondrial genetic markers, the duplex-PCR was used to find genetic traces of the introgression between the species in Hungarian populations.

## Materials and Methods

### Ethics statement

All procedures involving the handling and treatment of fish used for the study were approved by the Capital and Pest County Government Office for Food Chain Safety and Animal Health (Permit numbers: 22.1/527/003/2008 & XIV-I-001/2302-4/2012).

### Experimental fish and sample collection

As a genetic control of the collected samples and the species-specific markers, 20 genetic samples were examined relative to reference specimens for each of the three bullhead species (*Ameiurus nebulosus, Ameiurus melas*, and *Ameiurus natalis*) from native habitats (Fig. S1). Caudal fin clips were collected from fish in the USA; *Ameiurus melas* samples from the Highlands Park Pond, Edina, MN, USA (44°54′37.9″N, 93°22′02.1″W), *Ameiurus natalis* from Lake Bavaria, Victoria, MN, USA (44°50′12.7″N, 93°38′35.8″W), and *Ameiurus nebulosus* from Bush Lake, Bloomington, MN, USA (44°50′09.8″N, 93°23′03.2″W). All the samples were shipped and stored in 96% ethanol until further processing.

**Figure 1:**
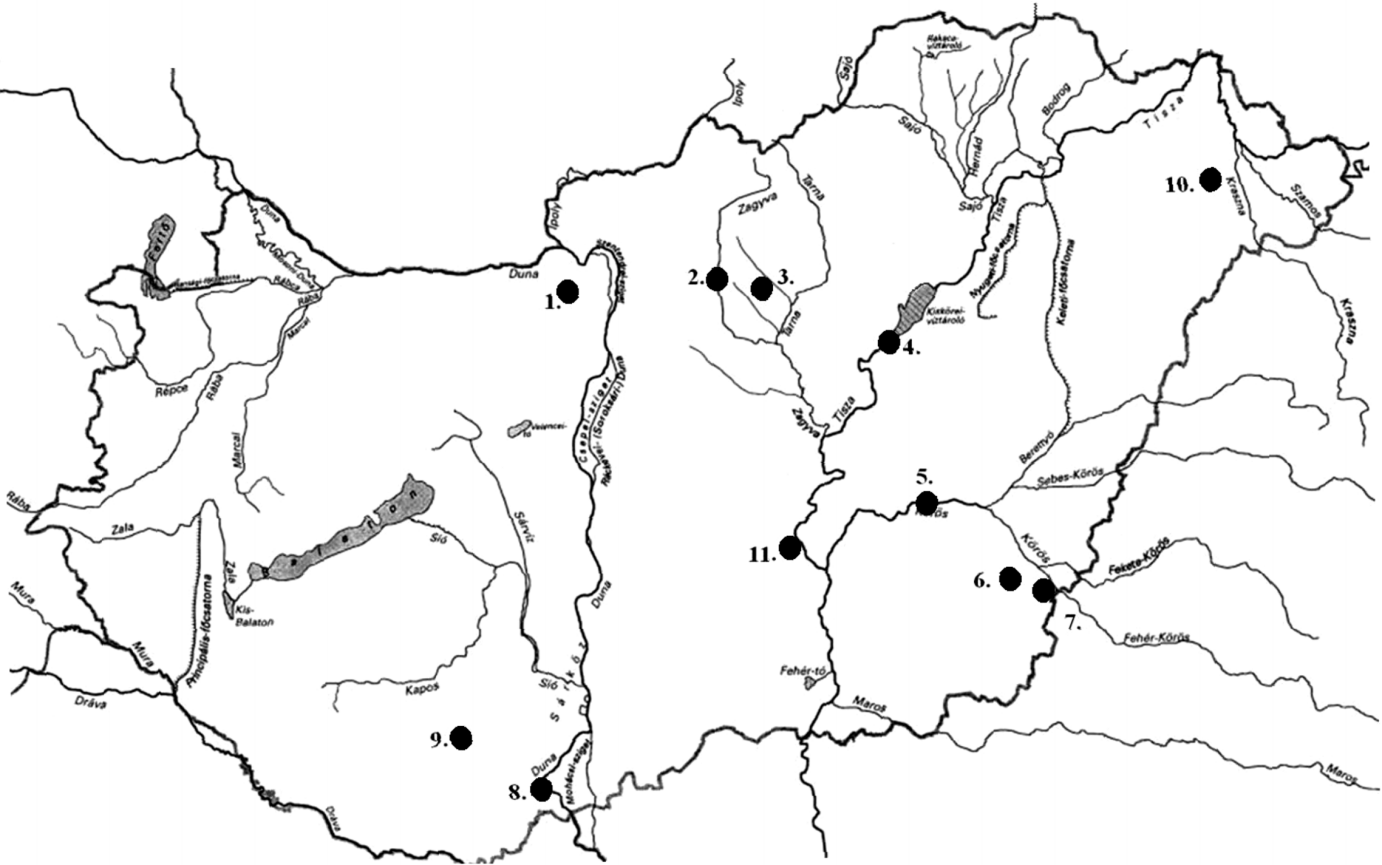
Sampling locations of bullhead catfishes in Hungary. Samples were collected from the following eleven locations: 1. Pilisvörösvár Lake; 2. Lőrinci Lake (Hatvan); 3. Adács Lake; 4. Jászság Canal; 5. River Hármas-Körös, Gyomaendrőd; 6. River Kettős-Körös, Békéscsaba; 7. River Körös, Dénesmajor; 8. Lake Külső-Béda, Mohács; 9. River Dráva, Majláthpuszta, Pécs; 10. Vaja Lake; 11. Szikra Backwater, Tőserdő. For the exact coordinates of locations, please see Table S1.

In the field experiment, 466 specimens were collected from 11 Hungarian drainage basins (without preliminary information on their identity) for molecular genetic examinations from the following lakes and rivers: the River Dráva, Majláthpuszta, Pécs, River Hármas-Körös, Gyomaendrőd, Vaja Lake, Pilisvörösvár Lake, Adács Lake, River Körös Dénesmajor, Jászság Canal, River Kettős-Körös Békéscsaba, Lőrinci Lake in Hatvan (connected to the River Zagyva), Lake Külső-Béda Mohács (connected to the Danube), and Szikra Backwater, Tőserdő. Figure 1 shows the locations of samples collection places, while Table S1 summarizes the number of the collected specimens and the GPS coordinates of the collecting places. For the DNA analyses, caudal fin clips were collected from each specimen and stored in absolute ethanol at −20 °C until further processing. All animal works were carried out after over-anesthetization of fish by clove oil (*Syzygium aromaticum*).

### DNA preparation

DNA samples were isolated from the caudal fin clips with optimized salt precipitation procedure (Miller, Dykes & Polesky, 1988). A total of 600 μl TAIL buffer (50 mM Tris–HCl, 10 mM EDTA, and 150 mM NaCl 1% SDS) was added to the cca. 1 cm^2^ fin sample in a 1.5-ml reaction tube, then 100 μg proteinase K enzyme (Fermentas) was used to digest the tissue. The mixture was incubated at 55 °C for 90 min with continuous shaking (200 rpm). To collect the undigested tissue debris, the tubes were centrifuged at room temperature for 15 min at 14,500 rpm. After that, 20 μg RNase A (Fermentas) was added to the supernatant and incubated for 20 min at room temperature. Then, 300 μl oversaturated (5–6 M) NaCl and 30 μl 7.5 M NH_4_Ac (ammonium acetate) were added to the mixture. After 1 min incubation at room temperature, the samples were centrifuged for 3 min (14,500 rpm), then 400 μl −20 °C isopropyl alcohol was added and mixed gently. After centrifugation (10 min, 4 °C, 14,500 rpm), the DNA pellet was washed with 300 μl 70% ethanol. The extracted DNA was dissolved in 50 μl TE (10 mM Tris, pH: 7.5) buffer. Before use, the samples were quantified by NanoPhotometer spectrophotometry (IMPLAN GmbH.) and diluted to a concentration of 50 ng/μl by TE buffer and stored at −20 °C.

### Identification of species-specific genetic markers

During the adaptation of different mitochondrial primer pairs (Ivanova et al., 2007; Xiao, Zhang & Liu, 2001; Bottero et al., 2007; Jondeung, Sangthong & Zardoya, 2007; Palumbi, 1996) an additional bands were amplified from *Ameiurus nebulosus* and *Ameiurus melas* samples by *cytochrome b*-specific primers (CytB_L_14724F and Cytbasa_R; Table 1) that annealed close to the targeted amplicon. As the size of these fragments was quite different between the two species and they were used to develop species-specific genetic markers. Amplifications were performed in 50 μl reaction mixture, which contained 1X reaction buffer (Fermentas) 0.4 mM dNTP, 0.25 μM primer, 1.5 mM MgCl_2_, 1 U Taq polymerase, and 200 ng template DNA. PCR profile consisted of initial denaturation for 1 min at 94 °C followed by 12 cycles of pre-amplification (denaturation at 94 °C for 20 s, annealing at 50 °C, and extension at 68 °C for 9 min) and 21 cycles of amplification (denaturation at 94 °C for 20 s, annealing at 50 °C, and extending at 68 °C for 9 min + 15 s/cycle). The final extension at 72 °C was performed for 5 min. In order to detect the PCR product, 1.5% agarose gel electrophoresis, containing 0.5 μg/ml ethidium bromide was used. The resulting patterns were photographed under UV-lamp and analyzed visually.

**Table 1:** List of used oligoes. Primer names, sequences, the appropriate product sizes and the origin of the oligos.

Primer name		Sequence	Product size	Origin of the oligos
1.	FF2d	TTCTCCACCAACCACAARGAYATYGG	708 bp	Ivanova et al. (2007)
2.	FR1d	CACCTCAGGGTGTCCGAARAAYCARAA		
3.	CytB_L_14724F	GACTTGAAAACCCACCGTTG	*Ameiurus nebulosus* 925 bp *Ameiurus melas* 791 bp all bullhead species 421 bp	Xiao, Zhang & Liu (2001)
4.	Cytbasa_R	GAAGGCGGTCATCATAACTAG		Bottero et al. (2007)
5.	Neb_F	CTGCTACATGCTAAGGCTAACG	*Ameiurus nebulosus* 591 bp *Ameiurus melas* 457 bp	this study
6.	Neb_R	GGATTATTGTGGCGATTGGT		this study
7.	P5	CCGAACTTAAGTTATAGCTGGTTGC	1,175 bp	Jondeung, Sangthong & Zardoya (2007)
8.	16sarL 59	CGCCTGTTTATCAAAAACAT		Palumbi (1996)
9.	New-Sp6	AGCTATTTAGGTGACACTATAG	Seq	Sambrook & Russell (2001)
10.	T7	TAATACGACTCACTATAGGG	Seq	

**Notes:**

1–2 are universal primers for fish Cytochrome Oxidase I (COI); 3–4 are universal primers for Cytochrome B; 5–8 oligoes for species-specific multiplex of *Ameiurus* spp.; 9–10 are the used sequencing primers.

The amplified extra bands were excised from agarose gel and isolated by QIA quick Gel Extraction Kit *(Qiagen)*, following the manufacturer’s recommendations. Each fragment was ligated into plasmid vector using pGEM-T Easy Vector System (Promega, Madison, WI, USA) and transformed into XL1 Blue *Escherichia coli* cells (Stratagene, San Diego, CA, USA). Several clones containing an insert of the right size for every fragment were sequenced on both strands (Fig. S1).

### Mitochondrial DNA analysis

The universal FF2d and FR1d primers (Ivanova et al., 2007) were chosen for mitochondrial sequence-based species identification. These primers amplify a 708 bp long section of the *cytochrome oxidase I* gene (COI) (Table 1; Fig. S1).

Amplifications were performed in 50 μl reaction mixture, which contained 1X reaction buffer (Fermentas) 0.4 mM dNTP, 0.250 μM primers, 1.5 mM MgCl_2_, 1 U Taq polymerase, and 200 ng template DNA. PCR profiles consisted of initial denaturation for 1 min at 94 °C followed by 37 cycles of amplification (denaturation at 94 °C for 15 s, annealing at 55 °C for 20 s, and extending at 72 °C for 2 min). The final extension at 72 °C was performed for 5 min. PCR products were assessed by 1.5% agarose gel electrophoresis and sequenced.

### Sequencing of PCR products

The PCR-amplified mitochondrial COI fragments and species-specific fragment-containing plasmids were cleaned with NucleoSpin Gel and PCR Clean-up (Macherey-Nagel) and High speed plasmid mini (Geneaid) kits before sequencing with FF2d, New-S6 or T7 primers (Table 1) and BigDye Terminator Sequencing Kit (ABI) following the manufacturer’s recommendations (Fig. S1). The sequences were determined on ABI Prism 3130 (ABI) sequencer with POP7 polymer. Sequences were analyzed using FinchTV 1.4 (Geospiza website); Mega5 (Tamura et al., 2011) or CLUSTAL 2.1 (Larkin et al., 2007) software. The sequences were also used to search GenBank using standard nucleotide BLAST on nr/nt nucleotide collection with megablast algorithm (http://www.ncbi.nlm.nih.gov/blast).

### Multiplex PCR reaction for species identification

Specific multiplex PCR reactions were set up and optimized as recommended by Henegariu et al. (1997) for identification of *Ameiurus nebulosus, Ameiurus melas* and their hybrids based on consensus flanking sequences of the previously identified species-specific amplicons and the mitochondrial 16S rDNA gene. A specific primer pair (Neb_F, Neb_R; Table 1) that amplified different size of genomic fragments from the two species was designed with Primer Express 3.0.1 software (ABI). In addition, a control (P5 and 16sarL 59 for amplification of partial sequence of 16S rDNS gene) primer pair that amplified the same fragment size from all three species was also used in the multiplex reaction as a positive control. The annealing temperature, the duration of elongation as well as the concentration of additives and the two primer pairs were varied until all three products were amplified to similar band intensity from mixed DNA of *Ameiurus nebulosus* and *Ameiurus melas*.

The optimized master mix for the multiplex PCR contained: 1X reaction buffer (Fermentas); 0.4 mM dNTP; 90 pM P5 and 16sarL 59 primers and 125 pM Neb_F and Neb_R primers; 1.5 mM MgCl_2_; 0.1% Tween20; 4% Dimethyl Sulfoxide; 0,375 ng Bovine serum albumin; 1.5 U Taq polymerase and 50 ng template DNA in 15 μl final volume.

Optimal cycling conditions for the multiplex amplification were: a pre-amplification denaturation at 95 °C for 10 min, followed by two cycles of 95 °C for 15 s, 56 °C for 60 s, and 72 °C for 140 s, then 35 cycles of 95 °C for 15 s, 56 °C for 20 s, and 72 °C for 2 min and a final extension of 72 °C for 5 min using a Mastercycler (Eppendorf) PCR machine. Products were separated and analyzed as described above. The specificity of the multiplex PCRs was confirmed by the “genetically pure” individual samples from the native habitats (Fig. S1).

## Results

### Identification and characterization of new species-specific genomic DNA markers from *Ameiurus melas* and *Ameiurus nebulosus*

Several published primer pairs (Ivanova et al., 2007; Xiao, Zhang & Liu, 2001; Bottero et al., 2007; Jondeung, Sangthong & Zardoya, 2007; Palumbi, 1996) were tested for amplification of different fragments of mitochondrial DNA from the three *Ameiurus* species. Two primers (CytB_L_14724F and Cytbasa_R) in addition to the universal 421 bp *cytochrome b*-specific fragment that was present in the PCR product of all three species, also amplified an additional band from *Ameiurus nebulosus* and *Ameiurus melas* samples (Fig. S2). The size of this additional fragment was considerably different in the two species: approximately 950 bp for *Ameiurus nebulosus* and nearly 800 bp for *Ameiurus melas*. This “second fragment” could be amplified from all of the 20 *Ameiurus nebulosus* and 20 *Ameiurus melas* samples from the original American habitat, but not from the 20 *Ameiurus natalis* samples, where only the universal (*cytochrome b*-specific) product could be detected (Fig. S2).

The full-length nucleotide sequence of the two species-specific fragments was determined and deposited in GenBank. The comparative sequence analysis of the fragments showed that they were amplified from the ortholog of the same sequence, but the variant found in *Ameiurus melas* was shorter.

**Figure 2:**
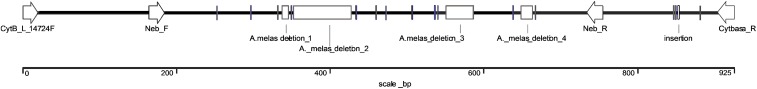
Schematic illustration of the comparison of species-specific sequences from *Ameiurus nebulosus* and *Ameiurus melas*. The arrows indicate the annealing locations of the original and our species-specific primers. The clear boxes show the deletions, the gray bars are single base pair substitutions or the 1 bp insertion in *Ameiurus melas* sequence compared to *Ameiurus nebulosus*.

The exact length of the *Ameiurus nebulosus-*derived sequence was 925 bp, (KX943306) while that of the *Ameiurus melas*-derived sequence was 791 bp (KX943305). The latter contained a 7-bp-, a 77-bp-, a 36-bp-, and a 15-bp-long deletion, as well as 14 single base pair substitutions and a 1-bp-long insertion compared to the *Ameiurus nebulosus* sequence (Figs. 2 and S3). The AT/(AT+GC) ratio was ca. 57% for both of the sequences. The alignment of the two sequences showed high sequence homology despite the difference in size (Fig. S3).

At the DNA level, neither of the markers showed significant homology with known sequences in GenBank. At the protein level, both sequences (147–320 bp region) showed very weak similarities with two nuclear genes: 36% homology was found with tsetse fly (*Glossina morsitans*) glutathione S-transferase and 33% homology with *Rhodnius prolixus* (689–796 bp region) putative spermidine proteins (Alves-Silva et al., 2010; Ribeiro et al., 2014).

### Development of a PCR-based marker system for the identification of *Ameiurus melas*, *Ameiurus nebulosus* and their F1 hybrids

**Figure 3:**
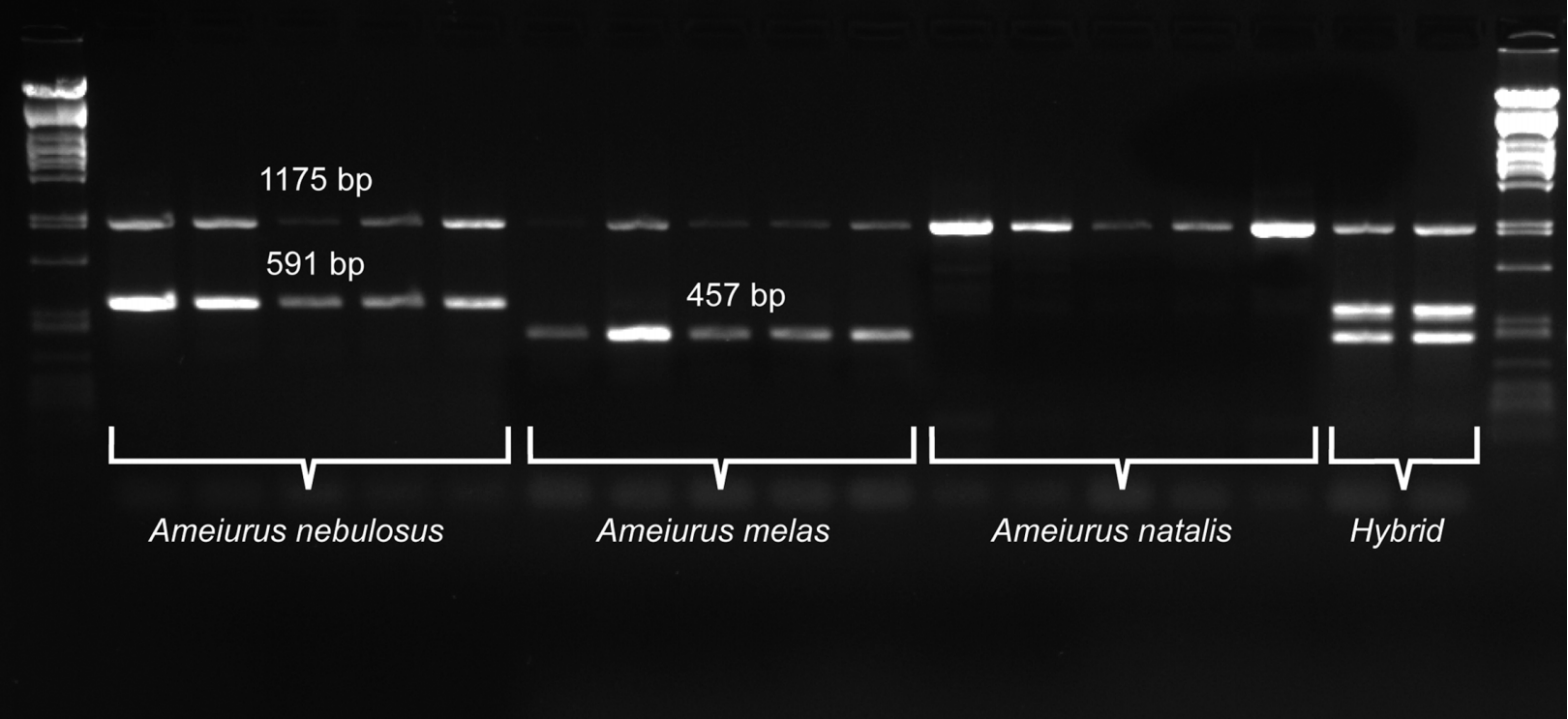
Duplex PCR-based molecular identification of three bullhead species: *Ameiurus nebulosus*, *Ameiurus melas*, and *Ameiurus natalis*. The PCR amplifies a control fragment (1,175 bp) from all samples, a 591-bp species-specific fragment from the *Ameiurus nebulosus* samples and a 457-bp amplicon from the *Ameiurus melas* samples. Both species-specific fragments are amplified from the hybrid individuals. The first and the last lanes contain Lambda *Pst*I molecular weight marker.

For a more precise identification of the two species and their hybrid identification, a new primer pair that annealed to the consensus region of the two species-specific sequences was developed (Neb_F and Neb_R; Table 1; Fig. 2). Similarly to the above pair, this primer pair also amplified an additional fragment of different size from both species: 591 bp from *Ameiurus nebulosus* and 457 bp from *Ameiurus melas*. In addition, it also amplified both fragments from hybrid genomes (Fig. 3).

Besides the above species-specific markers, several mitochondrial primers were tested as possible controls for a multiplex PCR. Finally, one primer pair was selected (P5 and 16sarL 59 primers; Table 1) that amplified a-1,175-bp-long fragment of the 16S rDNA gene. The optimized multiplex PCR amplified two bands from the original *Ameiurus melas* and *Ameiurus nebulosus* samples and three from their F1 hybrids, while only the control fragment was amplified from *Ameiurus natalis* (Fig. 3). However, this was confirmed only in the American samples.

### Mitochondrial sequence analysis reveal biased distribution of the two bullhead catfish species tested

Mitochondrial sequence analyses were performed for the identification of the species or their maternal lines. The segment of the *cytochrome oxidase I (COI)* gene was sequenced, and also used for barcoding (Ward et al., 2005; Kochzius et al., 2010). The universal oligos (FF2d & FR1d; Table 1) worked well in the examined *Ameiurus* species and specific fragments with the expected size (708 bp) were amplified. At least 25% of the available samples were sequenced from each habitat and it was complemented with those specimens, which showed the presence of the more rare *Ameiurus nebulosus* genomic DNA fragment (the number of determined sequences per habitat is summarized in Table S1) in the multiplex PCR. Altogether, we analyzed 176 individual samples, plus three specimens of every species from the American control samples. The sequences of the reference samples from North America were identical to the *Ameiurus melas*, *Ameiurus nebulosus*, and *Ameiurus natalis* sequences in GenBank, according to the BLAST results.

In total, 138 of the 176 (78.4%) Hungarian samples carried an *Ameiurus melas* mitochondrial sequence (Accession numbers: KX909375–KX909513; Table 2). Only three haplotypes could be found among these samples. In three sample (from Adács Lake–KX909378, Kettős Körös Békéscsaba–KX909397, and Lőrinci Lake–KX909444), the 50th position of the consensus sequence contained C instead of T and at 168th position contained G instead of A, while in a sample from River Kettős Körös Békéscsaba (KX909394) the 274th position contained G instead of A. The *Ameiurus melas* species was identified in every habitat. Only 38 (22.2%) examined sequences proved to originate from *Ameiurus nebulosus* (Accession numbers: KX909514–KX909551; Table 2). All of these sequences were of the same haplotype and were found in only three habitats; one specimen in the Canal of Jászság, 18 specimens in Vaja Lake, and 19 specimens in the Szikra Backwater.

**Table 2:** The results of species identification of *Ameiusus* spp. at different examined locations. The table shows the number of the identified genotypes with the species-specific genomic multiplex PCR and the mitochondrial Cytochrome Oxidase I sequencing at the different sampling locations.

	Places	Genotype					Contradiction of mitochondrial and genomic genotype
		Genomic DNA			Mitochondrial		
		*Ameiurus nebulosus*	*Ameiurus melas*	Hybrid	*Ameiurus nebulosus*	*Ameiurus melas*	
1.	Pilisvörösvár Lake	–	13	–	–	7	–
2.	Lőrinci Lake (Hatvan)	–	51	–	–	15	–
3.	Adács Lake	–	56	–	–	16	–
4.	Jászság Canal	–	6	–	1	5	1
5.	River Hármas-Körös Gyomaendrőd	–	8	–	–	8	–
6.	River Kettős-Körös Békéscsaba	–	11	–	–	9	–
7.	River Körös Dénesmajor	–	114	–	–	29	–
8.	Lake Külső-Béda Mohács	–	30	–	–	11	–
9.	River Dráva, Majláthpuszta, Pécs	–	52	–	–	14	–
10.	Vaja Lake	19	56	–	18	17	–
11.	Szikra Backwater, Tőserdő	19	29	2	19	7	1
	Total	38	426	2	38	138	2

### Molecular analysis of the spreading and hybridization of bullhead catfishes in Hungary

All of the available 466 samples were examined with the multiplex PCR test. According to the results, 426 specimens were *Ameiurus melas*, and 37 specimens were *Ameiurus nebulosus*, whereas only two specimens were hybrids at the Szikra Backwater, Tőserdő (Table 2; Line 11). *Ameiurus melas* was present in all habitats, but the *Ameiurus nebulosus* specific fragment pattern was found only at Vaja Lake (19 specimens; Table 2; Line 10) and the Szikra Backwater, Tőserdő (19 specimens; Table 2; Line 11).

The results of the multiplex PCR test and the mitochondrial genome sequencing were also compared individually (Table 2) and the two hybrid specimens had the *Ameiurus nebulosus* mitochondrial genotype, while one specimen from Szikra Backwater had *Ameiurus nebulosus* genomic and *Ameiurus melas* mitochondrial DNA, whereas one specimen from Jászság Canal had the opposite combination. All the hybrids appeared to be fertile as they had gonads with gametes. The occurrence of the *Ameiurus natalis* has not been recorded in Hungary so far.

## Discussion

Natural hybridization between closely related fish species is the consequence of adaptive invasion and the evolution of “superspecies” (Moran & Kornfield, 1993; Mallet, 2005). According to our results, this phenomenon can be observed between the bullhead catfishes in Europe. However, the identification of hybrid individuals is difficult. Since the evaluation of their external or internal morphological characters is ambiguous, the use of phenotypic analysis as the primary method of identification of species and hybrid specimens (Specziár, Bercsényi & Müller, 2009; Rutkayová et al., 2013) leads to a rather high chance of error and uncertainty, and sometimes it is difficult to evaluate individual parameters objectively.

A more reliable identification is possible with genetic species identification. Several marker types, including mitochondrial sequencing (Müller et al., 2010), alloenzyme (Poteaux, Berrebi & Bonhomme, 2000), PCR-RFLP (Müller et al., 2012), microsatellite (Lu, Basley & Bernatchez, 2001), SCAR and AFLP (Scotti-Saintagne et al., 2004), have been used successfully for hybrid detection. In the case of *Ameiurus nebulosus* and *Ameiurus melas*, the combination of morphological analysis and mitochondrial sequencing was utilized in the past (Hardman & Page, 2003; Hunnicutt, Cingolani & Voss, 2005). For more efficient identification, we developed a new multiplex PCR-based test.

Species-specific sequences were used to develop a multiplex PCR-based marker system for the identification of the *Ameiurus nebulosus* and *Ameiurus melas* species and their hybrids. This system amplifies a species-specific nuclear genomic fragment and a mitochondrial control (the nuclear genomic origin of the species-specific fragments are shown by the missing homology with the known *Ameiurus* mitochondrial genome sequences and by the codominant inheritance which were present in hybrid individuals). This genetic test works well both on North American and Hungarian samples. This multiplex PCR-based method proved to be user-friendly, rapid, and robust. The marker system was successfully used to identify hybrid specimens from natural populations and the combination of nuclear and mitochondrial markers increased the efficiency of hybrid detection compared to the nuclear genomic test.

When we analyzed the distribution of bullhead catfish species in Hungary, we found exclusively *Ameiurus melas* at most catchment areas. *Ameiurus nebulosus* was only detected at two of the 11 habitats, at the Szikra Backwater (Table 2, Line 11) and at Vaja Lake (Table 2, Line 10), where the two species (two populations) co-existed, despite the fact that it was the most eurytopic species before the second introduction of *Ameiurus melas* in the 1980s (Harka & Pintér, 1990; Wilhelm, 1999). The results confirm the hypothesis that *Ameiurus melas* has been spreading for decades and has gradually supplanted *Ameiurus nebulosus* in the natural waters of both Hungary and Europe (Elvira, 1984; Harka, 1997; Wilhelm, 1999; Koščo et al., 2000; Gante & Santos, 2002; Popa et al., 2006; Garcia-De-Lomas et al., 2009; Lusk, Luskova & Hanel, 2010; Nowak et al., 2010; Movchan, Talabishka & Velikopolskiy, 2014). As a note of caution, it must be mentioned that most descriptions of this spreading are based on phenotypic species identification (Harka & Pintér, 1990; Rutkayová et al., 2013).

Among the 466 individuals examined from the 11 populations, we found two hybrids based on the nuclear genomic DNA tests. These results confirmed the hypothesized hybridization of bullhead catfishes in Hungary (Harka & Pintér, 1990). Two additional cases of hybridization were detected based on a discrepancy between the results of the mitochondrial genome and genomic DNA tests as a sign for possible footprint of introgressive hybridization that might have happened two or more generations before. It indicates the fertility of the hybrid individuals. However, this phenomenon was shown; by less than 1% of the individuals and this level of hybridization is similar to that observed in other introgressive species (Mallet, 2005). Interestingly, in the putative hybrid zones of the North American continent the level of the bullhead catfishes hybridization were much higher (60 and 88%), based on discrepancy of PCR-RFLP analyses of mitochondrial and genomic DNA, but the utilized genomic markers do not show the presence of putative F1 hybrids (Walter et al., 2014). Probably, the offspring in the experiment originated from a single spawning of F2 or later generation of backcrossed hybrid individual.

In our study, the F1 hybrids carried the *Ameiurus nebulosus* mitochondrion, while all the North American identified hybrid individuals (41 specimens) carried *Ameiurus melas* mtDNA (Walter et al., 2014). This indicates that the hybridization can be happened in both directions of the sexes, because hybrid individuals were identified both with *Ameiurus melas* and *Ameiurus nebulosus* mtDNA. This phenomenon is present only in one-third of the hybridizing species (Wirtz, 1999). In Hungary, the hybrids were fertile (they had developed gonads) and they could have potentially bred with both of the two parental species (the discrepancies of the genomic and mtDNA were found in both combinations) (Harka & Pintér, 1990; this study).

Although the efficiency of the hybrid identification in later generations is restricted by random segregation and possible recombination of the chromosomes, our new marker system verifies the hybridization of invasive bullhead catfishes in Hungarian natural populations. Mathematical modeling of hybrid identification in later generations showed the necessity of data from more genomic markers. More than four markers are necessary for rough classification of hybrids, whereas, depending on the generation number, upward of 70 markers are required for reliable discrimination of the pure species and the backcrossed hybrids (Boecklen & Howard, 1997). The recently developed multiplex marker set can be complemented with the suitable nuclear recombination activating gene 2 (Rag2) based PCR-RFLP marker developed by others (Hunnicutt, Cingolani & Voss, 2005; Walter et al., 2014), but the number of the required discriminative markers makes robust identification of bullhead hybrids difficult. In order to confirm/test the sensibility and usability, such a marker set artificial production of F2 (or later generation) hybrids would be recommended. In species complexes, the separation of the genetic characters is decreasing in every generation due to the level of continuous hybridization, backcross, random segregation, and possible recombination of the chromosomes. The mixing of divergent genomes from different parental taxa can generate new genetic combinations leading to novel, transgressive phenotypes upon which selection can act (Fiss et al., 1997; Rieseberg, Archer & Wayne, 1999; Müller et al., 2010). Consequently, further investigations are needed to examine morphological and genetic characteristics of Fx hybrids.

## Supplemental Information

10.7717/peerj.2804/supp-1 Supplemental Information 1 Summary of the Hungarian bullhead samples. Exact geographic coordinates of sample collection locations and the number of catish samples collected per site. The number and percentage of the sequenced mitochondrial COI samples from the different sampling locations.

10.7717/peerj.2804/supp-2 Supplemental Information 2 The working strategy. Figure S1: A schematic flowchart of the used methods (SS mean species specific fragmets.).

10.7717/peerj.2804/supp-3 Supplemental Information 3 Figure S2: The species-specific fragment pattern amplified by the CytB_L_14724F and Cytbasa_R primers. Lanes from left: (1–9) *A. nebulosos*; (10–18) *A. melas*; (19–27) *A. natalis*; (28) molecular weight marker Lambda PstI.

10.7717/peerj.2804/supp-4 Supplemental Information 4 Figure S3: Comparative alignment of *A. nebulosus* and *A. melas* species-specific nuclear sequences. The gray color highlights the differences between the sequences.

10.7717/peerj.2804/supp-5 Supplemental Information 5 Raw data of the genomic and mitochondrial analysis of specimens.

## Additional Information and Declarations

### Field Study Permissions

The following information was supplied relating to field study approvals (i.e., approving body and any reference numbers): All procedures involving the handling and treatment of fish used for the study were approved by the Capital and Pest County Government Office for Food Chain Safety and Animal Health (Permit numbers: 22.1/527/003/2008 & XIV-I-001/2302-4/2012).

### DNA Deposition

The following information was supplied regarding the deposition of DNA sequences: Mitochondrial sequence accession numbers: Ameiurus nebulosus: KX909514–KX909551; Ameiurus melas: KX909375–KX909513. Species specific sequence accession numbers: Ameiurus nebulosus: KX943306; Ameiurus melas: KX943305.

### Data Deposition

The following information was supplied regarding data availability: The raw data has been supplied as Supplemental Dataset Files.
