# Supplementary material for: Species-specific markers provide molecular genetic evidence for natural introgression of bullhead catfishes in Hungary
Source: PeerJ. 2017 Feb 28;5:e2804. doi: 10.7717/peerj.2804 (PMC5333548; doi:10.7717/peerj.2804)
Supplement: Supplemental Information 1 — Exact geographic coordinates of sample collection locations and the number of catish samples collected per site. The number and percentage of the sequenced mitochondrial COI samples from the different sampling locations. [file peerj-05-2804-s001.pdf]

Supplementary Informations

Species specific markers provide molecular genetic evidence for natural introgression of bullhead catfishes in Hungary

Beatrix Béres[1*], Dóra Kánainé Sipos[1,2], Tamás Müller[1], Ádám Staszny[1], Milán Farkas[3], Katalin Bakos[1,2], László Orbán[1,4], Béla Urbányi[1], Balázs Kovács[1,2*]

[1]Department of Aquaculture, Szent István University, Gödöllő, Hungary, [2]Regional University Center of Excellence in Environmental Industry, Szent István University, Gödöllő, Hungary, [3]Department of Environmental Safety and Ecotoxicology, Institute of Aquaculture and Environmental Safety, Agricultural and Environment Faculty, Szent István University Gödöllő, Hungary, [4] Reproductive Genomics Group, Temasek Life Sciences Laboratory, 1 Research Link, National University of Singapore, Singapore

Table S1: Exact geographic coordinates of sample collection locations and the number of catish samples collected per site. The number and percentage of the sequenced mitochondrial COI samples from the different sampling locations.

Number	Catchment area	Coordinates	Samples collected (all three species combined)	Mitochondrial (COI) sequences	% of sequenced individuals
**1.**	Pilisvörösvár Lake	47°36'44.4"N 18°54'55.5"E	13	7	54
**2.**	Lőrinci Lake (Hatvan)	47°42'56.3"N 19°40'49.9"E	51	15	29
**3.**	Adács Lake	47°42'20.7"N 19°57'08.3"E	56	15	27
**4.**	Jászság Canal	47°30'42.9"N 20°29'37.3"E	6	6	100
**5.**	River Hármas-Körös Gyomaendrőd	46°56'30.2"N 20°48'12.8"E	8	8	100
**6.**	River Kettős-Körös Békéscsaba	46°44'35.9"N 21°11'21.6"E	11	9	82
**7.**	River Körös Dénesmajor	46°39'14.5"N 21°25'28.9"E	114	28	25
**8.**	Lake Külső-Béda Mohács (connected to the Danube)	45°55'52.2"N 18°46'07.3"E	30	11	37
**9.**	River Dráva, Majláthpuszta,Pécs	45°47'11.1"N 18°03'34.6"E	52	14	27
**10.**	Vaja Lake	47°58'46.7"N 22°09'09.8"E	75	35	47
**11.**	Szikra Backwater,Tőserdő	46°51'14.2"N 19°59'25.7"E	50	27	54
**Σ**			466	**175**	**38**
