# Supplementary figures and images for: Species-specific markers provide molecular genetic evidence for natural introgression of bullhead catfishes in Hungary
Source: PeerJ. 2017 Feb 28;5:e2804. doi: 10.7717/peerj.2804 (PMC5333548; doi:10.7717/peerj.2804)

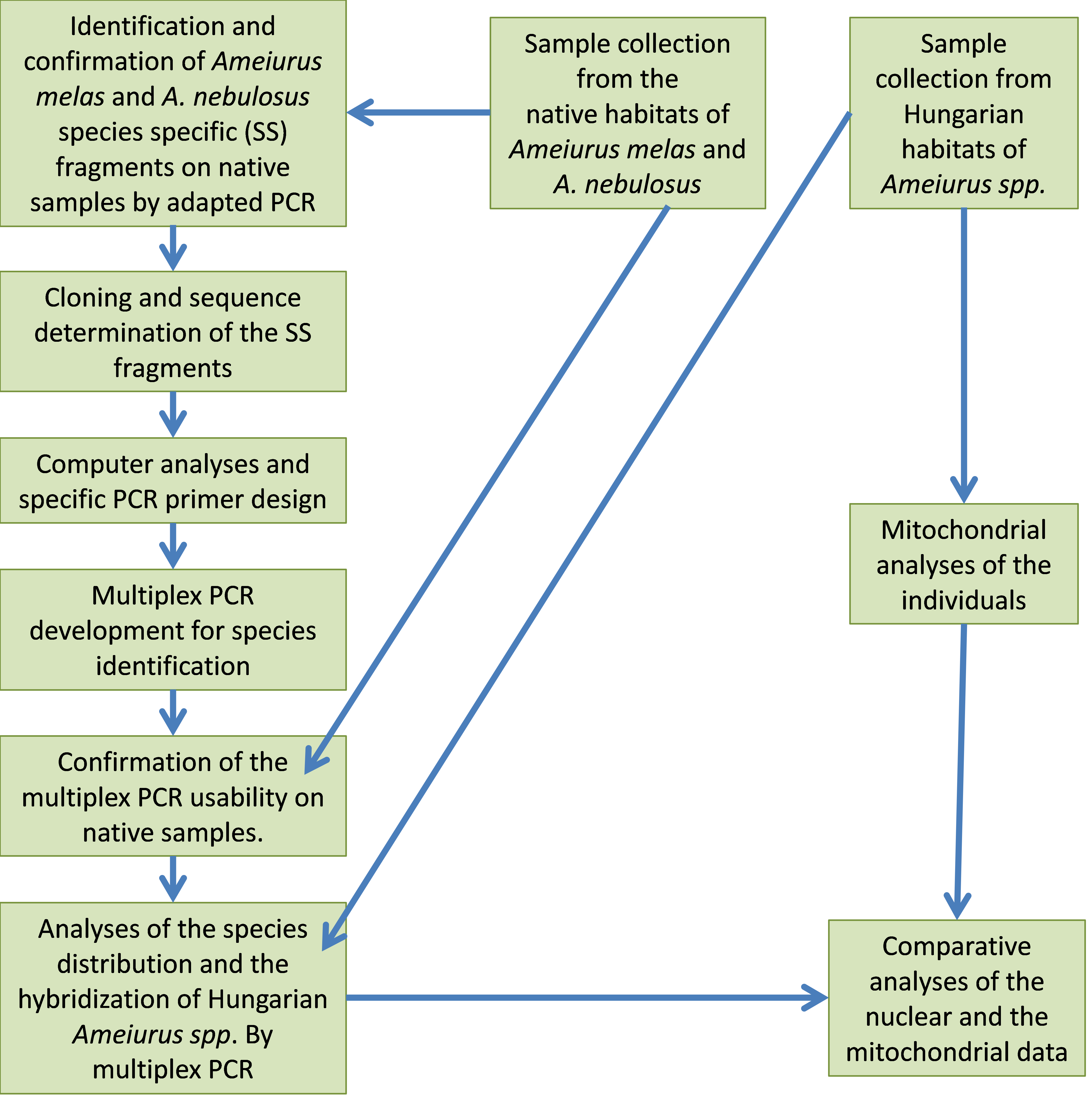

Supplement: Supplemental Information 2 — Figure S1: A schematic flowchart of the used methods (SS mean species specific fragmets.). [file peerj-05-2804-s002.png]

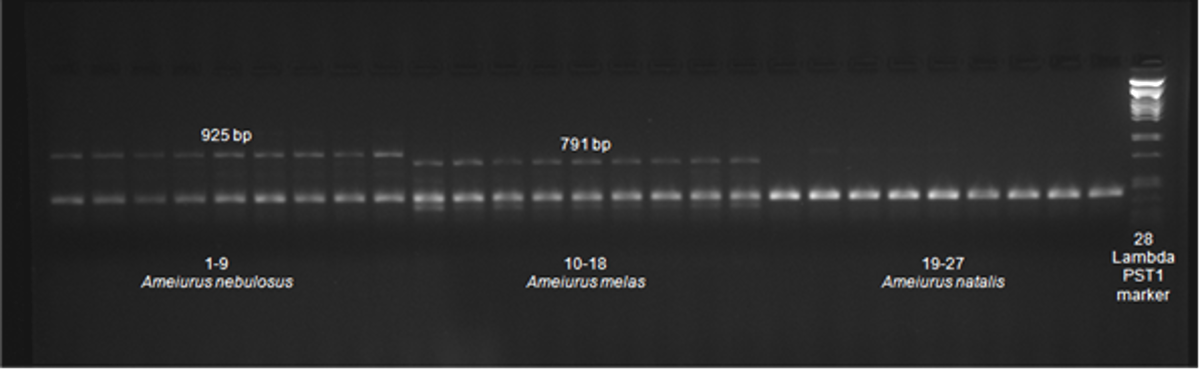

Supplement: Supplemental Information 3 — Lanes from left: (1–9) A. nebulosos; (10–18) A. melas; (19–27) A. natalis; (28) molecular weight marker Lambda PstI. [file peerj-05-2804-s003.png]

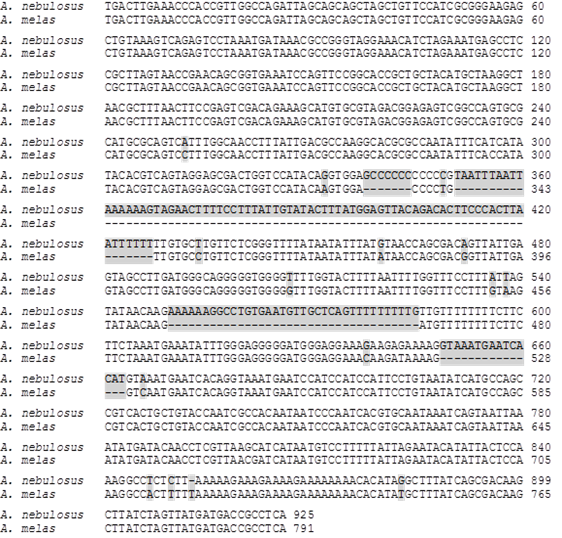

Supplement: Supplemental Information 4 — The gray color highlights the differences between the sequences. [file peerj-05-2804-s004.png]
