# Supplementary material for: Species-specific markers provide molecular genetic evidence for natural introgression of bullhead catfishes in Hungary
Source: PeerJ. 2017 Feb 28;5:e2804. doi: 10.7717/peerj.2804 (PMC5333548; doi:10.7717/peerj.2804)
Supplement: Supplemental Information 5 [file peerj-05-2804-s005.pdf]

Species specific markers provide molecular genetic evidence for natural introgression of bullhead catfishes in Hungary

Beatrix Béres1*, Dóra Kánainé Sipos1,2, Tamás Müller1, Ádám Staszny1, Milán Farkas3, Katalin Bakos1,2,László Orbán1,4, Béla Urbányi1, Balázs Kovács1,2*

1Department of Aquaculture, Szent István University, Gödöllő, Hungary, 2Regional University Center of Excellence in Environmental Industry, Szent István University, Gödöllő, Hungary, 3Department of Environmental Safety and Ecotoxicology, Institute of Aquaculture and Environmental Safety, Agricultural and Environment Faculty, Szent István University Gödöllő, Hungary, 4 Reproductive Genomics Group, Temasek Life Sciences Laboratory, 1 Research Link, National University of Singapore, Singapore

ID	Locations	Species based on duplex PCR	Spaecies based on COI sequencing
TÖ_4	Pilisvörösvár tó	*A.melas*	*A.melas*
TÖ_5	Pilisvörösvár	*A.melas*	*A.melas*
TÖ_6	Pilisvörösvár	*A.melas*	*A.melas*
TÖ_7	Pilisvörösvár	*A.melas*	
TÖ_8	Pilisvörösvár	*A.melas*	*A.melas*
TÖ_9	Pilisvörösvár	*A.melas*	*A.melas*
TÖ_10	Pilisvörösvár	*A.melas*	
TÖ_11	Pilisvörösvár	*A.melas*	*A.melas*
TÖ_12	Pilisvörösvár	*A.melas*	
TÖ_13	Pilisvörösvár	*A.melas*	
TÖ_14	Pilisvörösvár	*A.melas*	*A.melas*
TÖ_15	Pilisvörösvár	*A.melas*	
TÖ_16	Pilisvörösvár	*A.melas*	
TÖ_1	Adács tározó	*A.melas*	
TÖ_2	Adács	*A.melas*	
TÖ_3	Adács	*A.melas*	
TÖ_17	Adács	*A.melas*	
TÖ_18	Adács	*A.melas*	
TÖ_19	Adács	*A.melas*	*A.melas*
TÖ_20	Adács	*A.melas*	
TÖ_21	Adács	*A.melas*	
TÖ_22	Adács	*A.melas*	
TÖ_23	Adács	*A.melas*	*A.melas*
TÖ_24	Adács	*A.melas*	
TÖ_25	Adács	*A.melas*	
TÖ_26	Adács	*A.melas*	
TÖ_27	Adács	*A.melas*	*nincs adat*
TÖ_28	Adács	*A.melas*	
TÖ_29	Adács	*A.melas*	*A.melas*
TÖ_30	Adács	*A.melas*	
TÖ_31	Adács	*A.melas*	
TÖ_32	Adács	*A.melas*	
TÖ_33	Adács	*A.melas*	
TÖ_34	Adács	*A.melas*	
TÖ_35	Adács	*A.melas*	*A.melas*
TÖ_36	Adács	*A.melas*	*A.melas*
TÖ_37	Adács	*A.melas*	
TÖ_38	Adács	*A.melas*	
TÖ_39	Adács	*A.melas*	*A.melas*
TÖ_40	Adács	*A.melas*	
TÖ_41	Adács	*A.melas*	
TÖ_42	Adács	*A.melas*	
TÖ_43	Adács	*A.melas*	
TÖ_44	Adács	*A.melas*	*A.melas*
TÖ_45	Adács	*A.melas*	

TÖ_46	Adács	*A.melas*	
TÖ_47	Adács	*A.melas*	*A.melas*
TÖ_48	Adács	*A.melas*	*A.melas*
TÖ_49	Adács	*A.melas*	
TÖ_50	Adács	*A.melas*	
TÖ_51	Adács	*A.melas*	*A.melas*
TÖ_52	Adács	*A.melas*	
TÖ_53	Adács	*A.melas*	*A.melas*
TÖ_54	Adács	*A.melas*	
TÖ_55	Adács	*A.melas*	
TÖ_56	Adács	*A.melas*	
TÖ_57	Adács	*A.melas*	
TÖ_58	Adács	*A.melas*	*A.melas*
TÖ_59	Adács	*A.melas*	*A.melas*
TÖ_60	Adács	*A.melas*	
TÖ_61	Adács	*A.melas*	
TÖ_62	Adács	*A.melas*	
TÖ_63	Adács	*A.melas*	
TÖ_64	Adács	*A.melas*	
TÖ_65	Adács	*A.melas*	
TÖ_66	Adács	*A.melas*	
TÖ_67	Adács	*A.melas*	*A.melas*
TÖ_68	Adács	*A.melas*	*A.melas*
TÖ_69	Adács	*A.melas*	
TÖ_70	Gyomaendrőd Hármas Körös	*A.melas*	*A.melas*
TÖ_71	Gyomaendrőd	*A.melas*	*A.melas*
TÖ_72	Gyomaendrőd	*A.melas*	*A.melas*
TÖ_73	Gyomaendrőd	*A.melas*	*A.melas*
TÖ_74	Gyomaendrőd	*A.melas*	*A.melas*
TÖ_75	Gyomaendrőd	*A.melas*	*A.melas*
TÖ_76	Gyomaendrőd	*A.melas*	*A.melas*
TÖ_77	Gyomaendrőd	*A.melas*	*A.melas*
TÖ_78	Dénesmajor	*A.melas*	
TÖ_79	Dénesmajor GyulaKörös	*A.melas*	
TÖ_80	Dénesmajor	*A.melas*	
TÖ_81	Dénesmajor	*A.melas*	
TÖ_82	Dénesmajor	*A.melas*	
TÖ_83	Dénesmajor	*A.melas*	
TÖ_84	Dénesmajor	*A.melas*	*A.melas*
TÖ_85	Dénesmajor	*A.melas*	*A.melas*
TÖ_86	Dénesmajor	*A.melas*	
TÖ_87	Dénesmajor	*A.melas*	
TÖ_88	Dénesmajor	*A.melas*	*A.melas*
TÖ_89	Dénesmajor	*A.melas*	
TÖ_90	Dénesmajor	*A.melas*	
TÖ_91	Dénesmajor	*A.melas*	
TÖ_92	Dénesmajor	*A.melas*	
TÖ_93	Dénesmajor	*A.melas*	*A.melas*
TÖ_94	Dénesmajor	*A.melas*	
TÖ_95	Dénesmajor	*A.melas*	
TÖ_96	Dénesmajor	*A.melas*	
TÖ_97	Dénesmajor	*A.melas*	*A.melas*
TÖ_98	Dénesmajor	*A.melas*	
TÖ_99	Dénesmajor	*A.melas*	
TÖ_100	Dénesmajor	*A.melas*	*A.melas*
TÖ_101	Dénesmajor	*A.melas*	*A.melas*
TÖ_102	Dénesmajor	*A.melas*	
TÖ_103	Dénesmajor	*A.melas*	
TÖ_104	Dénesmajor	*A.melas*	
TÖ_105	Dénesmajor	*A.melas*	
TÖ_106	Dénesmajor	*A.melas*	*A.melas*
TÖ_107	Dénesmajor	*A.melas*	*A.melas*
TÖ_108	Dénesmajor	*A.melas*	

TÖ_109	Dénesmajor	*A.melas*	
TÖ_110	Dénesmajor	*A.melas*	
TÖ_111	Dénesmajor	*A.melas*	*A.melas*
TÖ_112	Dénesmajor	*A.melas*	
TÖ_113	Dénesmajor	*A.melas*	
TÖ_114	Dénesmajor	*A.melas*	
TÖ_115	Dénesmajor	*A.melas*	
TÖ_116	Dénesmajor	*A.melas*	
TÖ_117	Dénesmajor	*A.melas*	*A.melas*
TÖ_118	Dénesmajor	*A.melas*	
TÖ_119	Dénesmajor	*A.melas*	
TÖ_120	Dénesmajor	*A.melas*	
TÖ_121	Dénesmajor	*A.melas*	*A.melas*
TÖ_122	Dénesmajor	*A.melas*	*A.melas*
TÖ_123	Dénesmajor	*A.melas*	
TÖ_124	Dénesmajor	*A.melas*	
TÖ_125	Dénesmajor	*A.melas*	*A.melas*
TÖ_126	Dénesmajor	*A.melas*	
TÖ_127	Dénesmajor	*A.melas*	
TÖ_128	Dénesmajor	*A.melas*	*A.melas*
TÖ_129	Dénesmajor	*A.melas*	
TÖ_130	Dénesmajor	*A.melas*	
TÖ_131	Dénesmajor	*A.melas*	
TÖ_132	Dénesmajor	*A.melas*	*A.melas*
TÖ_133	Dénesmajor	*A.melas*	
TÖ_134	Dénesmajor	*A.melas*	
TÖ_135	Dénesmajor	*A.melas*	
TÖ_136	Dénesmajor	*A.melas*	
TÖ_137	Dénesmajor	*A.melas*	
TÖ_138	Dénesmajor	*A.melas*	
TÖ_139	Dénesmajor	*A.melas*	*A.melas*
TÖ_140	Dénesmajor	*A.melas*	*A.melas*
TÖ_141	Dénesmajor	*A.melas*	
TÖ_142	Dénesmajor	*A.melas*	
TÖ_143	Dénesmajor	*A.melas*	
TÖ_144	Dénesmajor	*A.melas*	
TÖ_145	Dénesmajor	*A.melas*	
TÖ_146	Dénesmajor	*A.melas*	
TÖ_147	Dénesmajor	*A.melas*	
TÖ_148	Dénesmajor	*A.melas*	
TÖ_149	Dénesmajor	*A.melas*	*A.melas*
TÖ_150	Dénesmajor	*A.melas*	
TÖ_151	Jászsági-csatorna	*A.melas*	*A.melas*
TÖ_152	Jászsági-csatorna Tisza	*A.melas*	*A. nebulosus*
TÖ_153	Jászsági-csatorna	*A.melas*	*A.melas*
TÖ_154	Jászsági-csatorna	*A.melas*	*A.melas*
TÖ_155	Jászsági-csatorna	*A.melas*	*A.melas*
TÖ_156	Jászsági-csatorna	*A.melas*	*A.melas*
TÖ_157	Békéscsaba	*A.melas*	*A.melas*
TÖ_158	Békéscsaba Kettőskörös	*A.melas*	*A.melas*
TÖ_159	Békéscsaba	*A.melas*	*A.melas*
TÖ_160	Békéscsaba	*A.melas*	*A.melas*
TÖ_161	Békéscsaba	*A.melas*	*A.melas*
TÖ_162	Békéscsaba	*A.melas*	
TÖ_163	Békéscsaba	*A.melas*	*A.melas*
TÖ_164	Békéscsaba	*A.melas*	*A.melas*
TÖ_165	Békéscsaba	*A.melas*	*A.melas*
TÖ_166	Békéscsaba	*A.melas*	*A.melas*
TÖ_167	Békéscsaba	*A.melas*	
TÖ_168	Dénesmajor Fehér Körös folyó	*A.melas*	*A.melas*
TÖ_169	Dénesmajor Fehér Körös folyó	*A.melas*	
TÖ_170	Dénesmajor Fehér Körös folyó	*A.melas*	
TÖ_171	Dénesmajor Fehér Körös folyó	*A.melas*	

TÖ_172	Dénesmajor Fehér Körös folyó	*A.melas*	
TÖ_173	Dénesmajor Fehér Körös folyó	*A.melas*	
TÖ_174	Dénesmajor Fehér Körös folyó	*A.melas*	
TÖ_175	Dénesmajor Fehér Körös folyó	*A.melas*	
TÖ_176	Dénesmajor Fehér Körös folyó	*A.melas*	*A.melas*
TÖ_177	Dénesmajor Fehér Körös folyó	*A.melas*	
TÖ_178	Dénesmajor Fehér Körös folyó	*A.melas*	
TÖ_179	Dénesmajor Fehér Körös folyó	*A.melas*	
TÖ_180	Dénesmajor Fehér Körös folyó	*A.melas*	*A.melas*
TÖ_181	Dénesmajor Fehér Körös folyó	*A.melas*	
TÖ_182	Dénesmajor Fehér Körös folyó	*A.melas*	
TÖ_183	Dénesmajor Fehér Körös folyó	*A.melas*	*A.melas*
TÖ_184	Dénesmajor Fehér Körös folyó	*A.melas*	
TÖ_185	Dénesmajor Fehér Körös folyó	*A.melas*	
TÖ_186	Dénesmajor Fehér Körös folyó	*A.melas*	
TÖ_187	Dénesmajor Fehér Körös folyó	*A.melas*	
TÖ_188	Dénesmajor Fehér Körös folyó	*A.melas*	
TÖ_189	Dénesmajor Fehér Körös folyó	*A.melas*	
TÖ_190	Dénesmajor Fehér Körös folyó	*A.melas*	*A.melas*
TÖ_191	Dénesmajor Fehér Körös folyó	*A.melas*	
TÖ_192	Dénesmajor Fehér Körös folyó	*A.melas*	
TÖ_193	Dénesmajor Fehér Körös folyó	*A.melas*	
TÖ_194	Dénesmajor Fehér Körös folyó	*A.melas*	
TÖ_195	Dénesmajor Fehér Körös folyó	*A.melas*	
TÖ_196	Dénesmajor Fehér Körös folyó	*A.melas*	*A.melas*
TÖ_197	Dénesmajor Fehér Körös folyó	*A.melas*	
TÖ_198	Dénesmajor Fehér Körös folyó	*A.melas*	
TÖ_199	Dénesmajor Fehér Körös folyó	*A.melas*	
TÖ_200	Dénesmajor Fehér Körös folyó	*A.melas*	*A.melas*
TÖ_201	Dénesmajor Fehér Körös folyó	*A.melas*	
TÖ_202	Dénesmajor Fehér Körös folyó	*A.melas*	
TÖ_203	Dénesmajor Fehér Körös folyó	*A.melas*	
TÖ_204	Dénesmajor Fehér Körös folyó	*A.melas*	*A.melas*
TÖ_205	Dénesmajor Fehér Körös folyó	*A.melas*	
TÖ_206	Dénesmajor Fehér Körös folyó	*A.melas*	
TÖ_207	Dénesmajor Fehér Körös folyó	*A.melas*	
TÖ_208	Dénesmajor Fehér Körös folyó	*A.melas*	*A.melas*
TÖ_209	Pécs-Dráva	*A.melas*	
TÖ_210	Pécs-Dráva	*A.melas*	
TÖ_211	Pécs-Dráva	*A.melas*	*A.melas*
TÖ_212	Pécs-Dráva	*A.melas*	
TÖ_213	Pécs-Dráva	*A.melas*	*A.melas*
TÖ_214	Pécs-Dráva	*A.melas*	
TÖ_215	Pécs-Dráva	*A.melas*	*A.melas*
TÖ_216	Pécs-Dráva	*A.melas*	
TÖ_217	Pécs-Dráva	*A.melas*	*A.melas*
TÖ_218	Pécs-Dráva	*A.melas*	
TÖ_219	Pécs-Dráva	*A.melas*	
TÖ_220	Pécs-Dráva	*A.melas*	
TÖ_221	Pécs-Dráva	*A.melas*	
TÖ_222	Pécs-Dráva	*A.melas*	
TÖ_223	Pécs-Dráva	*A.melas*	
TÖ_224	Pécs-Dráva	*A.melas*	
TÖ_225	Pécs-Dráva	*A.melas*	
TÖ_226	Pécs-Dráva	*A.melas*	
TÖ_227	Pécs-Dráva	*A.melas*	*A.melas*
TÖ_228	Pécs-Dráva	*A.melas*	
TÖ_229	Pécs-Dráva	*A.melas*	
TÖ_230	Pécs-Dráva	*A.melas*	*A.melas*
TÖ_231	Pécs-Dráva	*A.melas*	
TÖ_232	Pécs-Dráva	*A.melas*	

TÖ_233	Pécs-Dráva	*A.melas*	
TÖ_234	Pécs-Dráva	*A.melas*	
TÖ_235	Pécs-Dráva	*A.melas*	
TÖ_236	Pécs-Dráva	*A.melas*	
TÖ_237	Pécs-Dráva	*A.melas*	
TÖ_238	Pécs-Dráva	*A.melas*	
TÖ_239	Pécs-Dráva	*A.melas*	
TÖ_240	Pécs-Dráva	*A.melas*	*A.melas*
TÖ_241	Pécs-Dráva	*A.melas*	
TÖ_242	Pécs-Dráva	*A.melas*	*A.melas*
TÖ_243	Pécs-Dráva	*A.melas*	
TÖ_244	Pécs-Dráva	*A.melas*	
TÖ_245	Pécs-Dráva	*A.melas*	
TÖ_246	Pécs-Dráva	*A.melas*	
TÖ_247	Pécs-Dráva	*A.melas*	
TÖ_248	Pécs-Dráva	*A.melas*	
TÖ_249	Pécs-Dráva	*A.melas*	
TÖ_250	Pécs-Dráva	*A.melas*	*A.melas*
TÖ_251	Pécs-Dráva	*A.melas*	*A.melas*
TÖ_252	Pécs-Dráva	*A.melas*	
TÖ_253	Pécs-Dráva	*A.melas*	*A.melas*
TÖ_254	Pécs-Dráva	*A.melas*	*A.melas*
TÖ_255	Pécs-Dráva	*A.melas*	*A.melas*
TÖ_256	Pécs-Dráva	*A.melas*	
TÖ_257	Pécs-Dráva	*A.melas*	*A.melas*
TÖ_258	Pécs-Dráva	*A.melas*	
TÖ_259	Pécs-Dráva	*A.melas*	
TÖ_260	Pécs-Dráva	*A.melas*	
TÖ_261	Lőrinci-Hatvan Zagyva	*A.melas*	
TÖ_262	Lőrinci-Hatvan	*A.melas*	
TÖ_263	Lőrinci-Hatvan	*A.melas*	
TÖ_264	Lőrinci-Hatvan	*A.melas*	*A.melas*
TÖ_265	Lőrinci-Hatvan	*A.melas*	
TÖ_266	Lőrinci-Hatvan	*A.melas*	
TÖ_267	Lőrinci-Hatvan	*A.melas*	*A.melas*
TÖ_268	Lőrinci-Hatvan	*A.melas*	
TÖ_269	Lőrinci-Hatvan	*A.melas*	*A.melas*
TÖ_270	Lőrinci-Hatvan	*A.melas*	*A.melas*
TÖ_271	Lőrinci-Hatvan	*A.melas*	
TÖ_272	Lőrinci-Hatvan	*A.melas*	
TÖ_273	Lőrinci-Hatvan	*A.melas*	*A.melas*
TÖ_274	Lőrinci-Hatvan	*A.melas*	
TÖ_275	Lőrinci-Hatvan	*A.melas*	
TÖ_276	Lőrinci-Hatvan	*A.melas*	
TÖ_277	Lőrinci-Hatvan	*A.melas*	
TÖ_278	Lőrinci-Hatvan	*A.melas*	*A.melas*
TÖ_279	Lőrinci-Hatvan	*A.melas*	
TÖ_280	Lőrinci-Hatvan	*A.melas*	
TÖ_281	Lőrinci-Hatvan	*A.melas*	
TÖ_282	Lőrinci-Hatvan	*A.melas*	*A.melas*
TÖ_283	Lőrinci-Hatvan	*A.melas*	
TÖ_284	Lőrinci-Hatvan	*A.melas*	
TÖ_285	Lőrinci-Hatvan	*A.melas*	
TÖ_286	Lőrinci-Hatvan	*A.melas*	
TÖ_287	Lőrinci-Hatvan	*A.melas*	*A.melas*
TÖ_288	Lőrinci-Hatvan	*A.melas*	
TÖ_289	Lőrinci-Hatvan	*A.melas*	
TÖ_290	Lőrinci-Hatvan	*A.melas*	
TÖ_291	Lőrinci-Hatvan	*A.melas*	
TÖ_292	Lőrinci-Hatvan	*A.melas*	*A.melas*
TÖ_293	Lőrinci-Hatvan	*A.melas*	
TÖ_294	Lőrinci-Hatvan	*A.melas*	
TÖ_295	Lőrinci-Hatvan	*A.melas*	

TÖ_296	Lőrinci-Hatvan	*A.melas*	
TÖ_297	Lőrinci-Hatvan	*A.melas*	*A.melas*
TÖ_298	Lőrinci-Hatvan	*A.melas*	
TÖ_299	Lőrinci-Hatvan	*A.melas*	
TÖ_300	Lőrinci-Hatvan	*A.melas*	
TÖ_301	Lőrinci-Hatvan	*A.melas*	*A.melas*
TÖ_302	Lőrinci-Hatvan	*A.melas*	
TÖ_303	Lőrinci-Hatvan	*A.melas*	*A.melas*
TÖ_304	Lőrinci-Hatvan	*A.melas*	
TÖ_305	Lőrinci-Hatvan	*A.melas*	
TÖ_306	Lőrinci-Hatvan	*A.melas*	*A.melas*
TÖ_307	Lőrinci-Hatvan	*A.melas*	*A.melas*
TÖ_308	Lőrinci-Hatvan	*A.melas*	
TÖ_309	Lőrinci-Hatvan	*A.melas*	*A.melas*
TÖ_310	Lőrinci-Hatvan	*A.melas*	
TÖ_311	Lőrinci-Hatvan	*A.melas*	
TÖ_312	Mohács-Külső Béda Duna	*A.melas*	*A.melas*
TÖ_313	Mohács-Külső Béda	*A.melas*	
TÖ_314	Mohács-Külső Béda	*A.melas*	*A.melas*
TÖ_315	Mohács-Külső Béda	*A.melas*	
TÖ_316	Mohács-Külső Béda	*A.melas*	*A.melas*
TÖ_317	Mohács-Külső Béda	*A.melas*	*A.melas*
TÖ_318	Mohács-Külső Béda	*A.melas*	*A.melas*
TÖ_319	Mohács-Külső Béda	*A.melas*	
TÖ_320	Mohács-Külső Béda	*A.melas*	
TÖ_321	Mohács-Külső Béda	*A.melas*	*A.melas*
TÖ_322	Mohács-Külső Béda	*A.melas*	
TÖ_323	Mohács-Külső Béda	*A.melas*	
TÖ_324	Mohács-Külső Béda	*A.melas*	
TÖ_325	Mohács-Külső Béda	*A.melas*	
TÖ_326	Mohács-Külső Béda	*A.melas*	
TÖ_327	Mohács-Külső Béda	*A.melas*	*A.melas*
TÖ_328	Mohács-Külső Béda	*A.melas*	
TÖ_329	Mohács-Külső Béda	*A.melas*	
TÖ_330	Mohács-Külső Béda	*A.melas*	
TÖ_331	Mohács-Külső Béda	*A.melas*	
TÖ_332	Mohács-Külső Béda	*A.melas*	
TÖ_333	Mohács-Külső Béda	*A.melas*	*A.melas*
TÖ_334	Mohács-Külső Béda	*A.melas*	
TÖ_335	Mohács-Külső Béda	*A.melas*	*A.melas*
TÖ_336	Mohács-Külső Béda	*A.melas*	
TÖ_337	Mohács-Külső Béda	*A.melas*	*A.melas*
TÖ_338	Mohács-Külső Béda	*A.melas*	
TÖ_339	Mohács-Külső Béda	*A.melas*	*A.melas*
TÖ_340	Mohács-Külső Béda	*A.melas*	
TÖ_341	Mohács-Külső Béda	*A.melas*	
TÖ_V_1	Vaja	*A.nebulosus*	*A.nebulosus*
TÖ_V_2	Vaja	*A.nebulosus*	*A.nebulosus*
TÖ_V_3	Vaja	*A.nebulosus*	*A.nebulosus*
TÖ_V_4	Vaja	*A.nebulosus*	*A.nebulosus*
TÖ_V_5	Vaja	*A.nebulosus*	*A.nebulosus*
TÖ_V_6	Vaja	*A.nebulosus*	*A.nebulosus*
TÖ_V_7	Vaja	*A.nebulosus*	*A.nebulosus*
TÖ_V_8	Vaja	*A.nebulosus*	*A.nebulosus*
TÖ_V_9	Vaja	*A.nebulosus*	
TÖ_V_10	Vaja	*A.nebulosus*	*A.nebulosus*
TÖ_V_11	Vaja	*A.nebulosus*	*A.nebulosus*
TÖ_V_12	Vaja	*A.nebulosus*	*A.nebulosus*
TÖ_V_13	Vaja	*A.melas*	
TÖ_V_14	Vaja	*A.melas*	
TÖ_V_15	Vaja	*A.melas*	*A.melas*
TÖ_V_16	Vaja	*A.melas*	
TÖ_V_17	Vaja	*A.melas*	

TÖ_V_18	Vaja	*A.melas*	*A.melas*
TÖ_V_19	Vaja	*A.melas*	
TÖ_V_20	Vaja	*A.melas*	*A.melas*
TÖ_V_21	Vaja	*A.melas*	
TÖ_V_22	Vaja	*A.melas*	
TÖ_V_23	Vaja	*A.melas*	
TÖ_V_24	Vaja	*A.melas*	*A.melas*
TÖ_V_25	Vaja	*A.melas*	
TÖ_V_26	Vaja	*A.melas*	*A.melas*
TÖ_V_27	Vaja	*A.melas*	
TÖ_V_28	Vaja	*A.melas*	*A.melas*
TÖ_V_29	Vaja	*A.melas*	
TÖ_V_30	Vaja	*A.melas*	
TÖ_V_31	Vaja	*A.melas*	
TÖ_V_32	Vaja	*A.nebulosus*	*A.nebulosus*
TÖ_V_33	Vaja	*A.melas*	
TÖ_V_34	Vaja	*A.melas*	
TÖ_V_35	Vaja	*A.melas*	*A.melas*
TÖ_V_36	Vaja	*A.melas*	
TÖ_V_37	Vaja	*A.melas*	
TÖ_V_38	Vaja	*A.melas*	*A.melas*
TÖ_V_39	Vaja	*A.melas*	
TÖ_V_40	Vaja	*A.melas*	
TÖ_V_41	Vaja	*A.melas*	*A.melas*
TÖ_V_42	Vaja	*A.melas*	
TÖ_V_43	Vaja	*A.melas*	
TÖ_V_44	Vaja	*A.melas*	*A.melas*
TÖ_V_45	Vaja	*A.melas*	
TÖ_V_46	Vaja	*A.melas*	
TÖ_V_47	Vaja	*A.melas*	
TÖ_V_48	Vaja	*A.melas*	*A.melas*
TÖ_V_49	Vaja	*A.melas*	
TÖ_V_50	Vaja	*A.melas*	*A.melas*
TÖ_V_51	Vaja	*A.melas*	
TÖ_V_52	Vaja	*A.melas*	
TÖ_V_53	Vaja	*A.melas*	
TÖ_V_54	Vaja	*A.melas*	*A.melas*
TÖ_V_55	Vaja	*A.melas*	
TÖ_V_56	Vaja	*A.melas*	
TÖ_V_57	Vaja	*A.melas*	
TÖ_V_58	Vaja	*A.melas*	*A.melas*
TÖ_V_59	Vaja	*A.melas*	
TÖ_V_60	Vaja	*A.melas*	
TÖ_V_61	Vaja	*A.melas*	*A.melas*
TÖ_V_62	Vaja	*A.melas*	
TÖ_V_63	Vaja	*A.melas*	
TÖ_V_64	Vaja	*A.nebulosus*	*A.nebulosus*
TÖ_V_65	Vaja	*A.nebulosus*	*A.nebulosus*
TÖ_V_66	Vaja	*A.nebulosus*	*A.nebulosus*
TÖ_V_67	Vaja	*A.nebulosus*	*A.nebulosus*
TÖ_V_68	Vaja	*A.nebulosus*	*A.nebulosus*
TÖ_V_69	Vaja	*A.melas*	
TÖ_V_70	Vaja	*A.melas*	
TÖ_V_71	Vaja	*A.melas*	
TÖ_V_72	Vaja	*A.nebulosus*	*A.nebulosus*
TÖ_V_73	Vaja	*A.melas*	*A.melas*
TÖ_V_74	Vaja	*A.melas*	
TÖ_V_75	Vaja	*A.melas*	*A.melas*
TÖ_T_1	Tőserdő- Szikrai víztározó	*A.nebulosus*	*A.nebulosus*
TÖ_T_2	Tőserdő- Szikrai víztározó	*A.nebulosus*	*A.nebulosus*
TÖ_T_3	Tőserdő- Szikrai víztározó	*A.nebulosus*	*A.nebulosus*
TÖ_T_4	Tőserdő- Szikrai víztározó	*A.nebulosus*	*A.nebulosus*
TÖ_T_5	Tőserdő- Szikrai víztározó	*A.nebulosus*	*A.nebulosus*

TÖ_T_6	Tőserdő- Szikrai víztározó	*A.nebulosus*	*A.nebulosus*
TÖ_T_7	Tőserdő- Szikrai víztározó	*A.nebulosus*	*A.nebulosus*
TÖ_T_8	Tőserdő- Szikrai víztározó	*A.nebulosus*	*A.nebulosus*
TÖ_T_9	Tőserdő- Szikrai víztározó	*HIBRID*	*A.nebulosus*
TÖ_T_10	Tőserdő- Szikrai víztározó	*A.nebulosus*	*A.nebulosus*
TÖ_T_11	Tőserdő- Szikrai víztározó	*A.nebulosus*	*A.nebulosus*
TÖ_T_12	Tőserdő- Szikrai víztározó	*A.nebulosus*	*A.nebulosus*
TÖ_T_13	Tőserdő- Szikrai víztározó	*A.nebulosus*	*A.nebulosus*
TÖ_T_14	Tőserdő- Szikrai víztározó	*A.nebulosus*	*A.nebulosus*
TÖ_T_15	Tőserdő- Szikrai víztározó	*A.nebulosus*	*A.nebulosus*
TÖ_T_16	Tőserdő- Szikrai víztározó	*A.nebulosus*	*A.nebulosus*
TÖ_T_17	Tőserdő- Szikrai víztározó	*A.nebulosus*	*A.melas*
TÖ_T_18	Tőserdő- Szikrai víztározó	*A.nebulosus*	*A.nebulosus*
TÖ_T_19	Tőserdő- Szikrai víztározó	*A.melas*	*A.melas*
TÖ_T_20	Tőserdő- Szikrai víztározó	*A.melas*	
TÖ_T_21	Tőserdő- Szikrai víztározó	*A.melas*	
TÖ_T_22	Tőserdő- Szikrai víztározó	*A.melas*	*A.melas*
TÖ_T_23	Tőserdő- Szikrai víztározó	*A.melas*	
TÖ_T_24	Tőserdő- Szikrai víztározó	*A.melas*	
TÖ_T_25	Tőserdő- Szikrai víztározó	*HIBRID*	*A.nebulosus*
TÖ_T_26	Tőserdő- Szikrai víztározó	*A.nebulosus*	*A.nebulosus*
TÖ_T_27	Tőserdő- Szikrai víztározó	*A.nebulosus*	*A.nebulosus*
TÖ_T_28	Tőserdő- Szikrai víztározó	*A.melas*	
TÖ_T_29	Tőserdő- Szikrai víztározó	*A.melas*	
TÖ_T_30	Tőserdő- Szikrai víztározó	*A.melas*	
TÖ_T_31	Tőserdő- Szikrai víztározó	*A.melas*	*A.melas*
TÖ_T_32	Tőserdő- Szikrai víztározó	*A.melas*	
TÖ_T_33	Tőserdő- Szikrai víztározó	*A.melas*	
TÖ_T_34	Tőserdő- Szikrai víztározó	*A.melas*	
TÖ_T_35	Tőserdő- Szikrai víztározó	*A.melas*	
TÖ_T_36	Tőserdő- Szikrai víztározó	*A.melas*	
TÖ_T_37	Tőserdő- Szikrai víztározó	*A.melas*	*A.melas*
TÖ_T_38	Tőserdő- Szikrai víztározó	*A.melas*	*A.melas*
TÖ_T_39	Tőserdő- Szikrai víztározó	*A.melas*	
TÖ_T_40	Tőserdő- Szikrai víztározó	*A.melas*	
TÖ_T_41	Tőserdő- Szikrai víztározó	*A.melas*	
TÖ_T_42	Tőserdő- Szikrai víztározó	*A.melas*	*A.melas*
TÖ_T_43	Tőserdő- Szikrai víztározó	*A.melas*	
TÖ_T_44	Tőserdő- Szikrai víztározó	*A.melas*	
TÖ_T_45	Tőserdő- Szikrai víztározó	*A.melas*	
TÖ_T_46	Tőserdő- Szikrai víztározó	*A.melas*	
TÖ_T_47	Tőserdő- Szikrai víztározó	*A.melas*	
TÖ_T_48	Tőserdő- Szikrai víztározó	*A.melas*	
TÖ_T_49	Tőserdő- Szikrai víztározó	*A.melas*	
TÖ_T_50	Tőserdő- Szikrai víztározó	*A.melas*	
		466	*176*
